# Long-read sequencing for reliably calling the *mompS* allele in *Legionella pneumophila* sequence-based typing

**DOI:** 10.3389/fcimb.2023.1176182

**Published:** 2023-05-15

**Authors:** Anne Vatland Krøvel, Marit A. K. Hetland, Eva Bernhoff, Anna Steensen Bjørheim, Markus André Soma, Iren H. Löhr

**Affiliations:** ^1^ Department of Medical Microbiology, Stavanger University Hospital, Stavanger, Norway; ^2^ National Reference Laboratory for Legionella, Stavanger University Hospital, Stavanger, Norway; ^3^ Department of Biological Sciences, Faculty of Mathematics and Natural Sciences, University of Bergen, Bergen, Norway; ^4^ Department of Clinical Science, Faculty of Medicine, University of Bergen, Bergen, Norway

**Keywords:** *Legionella pneumophila* SBT, mompS, WGS, Illumina, short-reads, ONT, long-reads

## Abstract

Sequence-based typing (SBT) of *Legionella pneumophila* is a valuable tool in epidemiological studies and outbreak investigations of Legionnaires’ disease. In the *L. pneumophila* SBT scheme, *mompS2* is one of seven genes that determine the sequence type (ST). The *Legionella* genome typically contains two copies of *mompS (mompS1* and *mompS2).* When they are non-identical it can be challenging to determine the *mompS2* allele, and subsequently the ST, from Illumina short-reads. In our collection of 233 *L. pneumophila* genomes, there were 62 STs, 18 of which carried non-identical *mompS* copies. Using short-reads, the *mompS2* allele was misassembled or untypeable in several STs. Genomes belonging to ST154 and ST574, which carried *mompS1* allele 7 and *mompS2* allele 15, were assigned an incorrect *mompS2* allele and/or *mompS* gene copy number when short-read assembled. For other isolates, mainly those carrying non-identical *mompS* copies, short-read assemblers occasionally failed to resolve the structure of the *mompS-*region, also resulting in untypeability from the short-read data. In this study, we wanted to understand the challenges we observed with calling the *mompS*2 allele from short-reads, assess if other short-read methods were able to resolve the *mompS*-region, and investigate the possibility of using long-reads to obtain the *mompS* alleles, and thereby perform *L. pneumophila* SBT from long-reads only. We found that the choice of short-read assembler had a major impact on resolving the *mompS*-region and thus SBT from short-reads, but no method consistently solved the *mompS2* allele. By using Oxford Nanopore Technology (ONT) sequencing together with Trycycler and Medaka for long-read assembly and polishing we were able to resolve the *mompS* copies and correctly identify the *mompS2* allele, in accordance with Sanger sequencing/EQA results for all tested isolates (n=35). The remaining six genes of the SBT profile could also be determined from the ONT-only reads. The STs called from ONT-only assemblies were also consistent with hybrid-assemblies of Illumina and ONT reads. We therefore propose ONT sequencing as an alternative method to perform *L. pneumophila* SBT to overcome the *mompS* challenge observed with short-reads. To facilitate this, we have developed ONTmompS (https://github.com/marithetland/ONTmompS), an *in silico* approach to determine *L. pneumophila* ST from long-read or hybrid assemblies.

## 1 Introduction

The *Legionella*-bacteria can cause a severe and potentially fatal form of pneumonia called Legionnaires’ disease (LD). When the bacteria colonize and multiply in man-made systems with favorable conditions for growth, it may pose a threat to human health, through inhalation of bacteria-contaminated aerosols (Bartram et al., 2007; Whiley et al., 2014). There are more than 60 known species of *Legionella* with varying pathogenicity (Parte et al., 2020). *Legionella pneumophila* is the species implicated in at least 90% of the reported LD cases worldwide (reviewed in Herwaldt and Marra, 2018; Chauhan and Shames, 2021). *L. pneumophila* can be subtyped into at least 15 serogroups based on surface molecules and also into sequence types (STs) determined by the seven genes *flaA, pilE, asd, mip, mompS, proA* and *neuA* (Gaia et al., 2005; Ratzow et al., 2007). The current number of defined STs is over 3100 (Legionella-SBT, 2023).

Sequence-based typing (SBT) is a valuable tool for source investigations and epidemiological studies of *L. pneumophila* that allows for rapid molecular typing and inter-laboratorial comparison (Gaia et al., 2005; Ratzow et al., 2007). Although *L. pneumophila* SBT performed by Sanger sequencing is still considered the gold standard, more recently, whole-genome sequencing (WGS) has become the method of choice, providing both ST and superior information about genetic relatedness, and is today indispensable in surveillance and outbreak investigations (Moran-Gilad et al., 2015; Khodr et al., 2016; Raphael et al., 2016; Krøvel et al., 2022; Ricci et al., 2022). Analyses like core or whole genome multi locus sequence typing (cgMLST/wgMLST) and single nucleotide polymorphism (SNP) analysis have higher discriminatory power than SBT and would provide more information to aid the identification of the source of an outbreak. 

**Figure 1:**
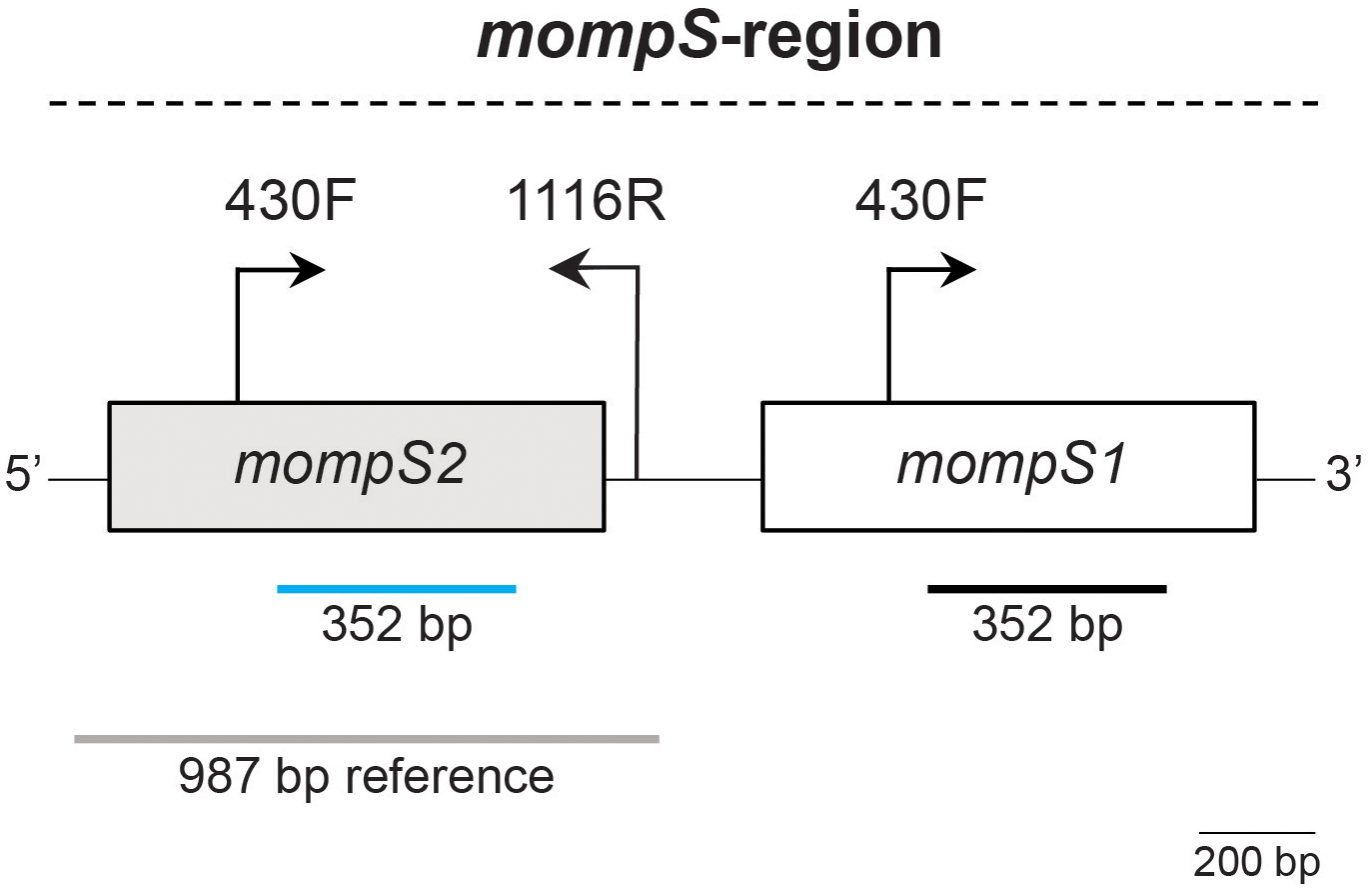
The *mompS*-region. The two *mompS* copies are closely positioned in the genome. The first step of Sanger sequencing uses primers 430F and 1116R to specifically amplify *mompS2* (Gaia et al., 2005; Gordon et al., 2017). The 352 bp conserved region used for determination of *mompS*2 in *L. pneumophila* SBT is marked by a blue line. The *mompS1* gene also contains the primer binding site for primer 430F and a 352 bp conserved region, marked by a black line. A reference sequence defined by Gordon et al. (2017) to distinguish *mompS2* from *mompS1*, which is also used in our ONTmompS *in silico* approach, is indicated by a grey line. The 987 bp reference sequence covers the 5’ flanking region from position 157, the entire coding sequence of *mompS*2 (204-1070) and the 3’ flanking region including primer 1116R. The positions refer to those in the GenBank sequence AF078136, which was used to design the original primers for Sanger sequencing. A 200 bp scalebar is indicated.

A challenge in the *L. pneumophila* SBT scheme is that the *mompS* gene, which is used to determine the ST, is usually present with two copies in the genome, *mompS1* and *mompS2*. Only *mompS2* contributes to the ST (Gaia et al., 2005; Gordon et al., 2017). The two *mompS* copies are closely positioned in the genome, resulting in a *mompS*-region of about 2100 bp (see **Figure 1**). *L. pneumophila* SBT by Sanger sequencing amplifies *mompS2* using specific primers. Short-read WGS gives a maximum of 300 bp reads, which are too short to cover the *mompS* gene let alone the entire *mompS*-region. When the two *mompS* copies are non-identical, existing *in silico* approaches for *L. pneumophila* SBT have been shown to have limitations, related to erroneous calling of the *mompS* alleles or incorrect assembly of the *mompS*-region, which may result in incorrect ST determination and/or untypeabilty (Gordon et al., 2017).

Illumina short-read WGS has so far been the method of choice for genomic surveillance because of the higher basecalling accuracy compared to long-read sequencing technologies like Oxford Nanopore Technologies (ONT). However, challenges with untypeability and resolution of repetitive regions due to the short read length has led to exploration of long-read sequencing as an alternative (Ben Khedher et al., 2022). At the moment, hybrid-assemblies of short- and long-reads are considered the WGS gold standard over ONT-only assemblies with regards to accuracy (Wick et al., 2023).

In our laboratory, we have observed several challenges related to calling *mompS* from short-reads. In diagnostics and surveillance of *Legionella*, time and cost is of the essence and generation of hybrid assemblies for all samples is not likely to be standard procedure. Therefore, the possibility of using ONT-only data to overcome the shortcomings observed in *L. pneumophila* SBT from short-reads is highly relevant and has to our knowledge not previously been investigated. In this study, we wanted to understand the challenges we observed using short-reads, assess if other short-read methods were able to resolve the *mompS*-region and investigate the possibility of using long-reads to obtain the *mompS* allele, and thereby perform *L. pneumophila* SBT from long-reads only.

## 2 Materials and methods

### 2.1 Sample collection

At the National Reference Laboratory for *Legionella*, Stavanger University Hospital, Norway, we have a collection of Illumina sequenced clinical and environmental isolates, collected between 2001 and 2022 (n=233). Of these, 35 were selected for further analysis in this study, based on discrepancies between the *mompS* result from Illumina short-read and Sanger sequencing, presence of non-identical *mompS* copies or untypeability of *mompS* (n=27). Isolates with identical copies of *mompS* were included as controls (n=8).

A further 46 isolates were included later in the study to validate the ONTmompS *in silico* SBT approach that we developed for assigning *L. pneumophila* ST (see section 3.3).

### 2.2 Whole-genome sequencing

The 35 *L. pneumophila* isolates had previously been Illumina short-read sequenced and the fastq-files deposited in our in-house sequence database. In short, DNA had been extracted using MagNA Pure 96 with the Pathogen Universal 200 4.0 purification protocol (Roche Applied Science, Penzberg, Germany). Genomic libraries were prepared using Illumina DNA library prep and sequenced to ≥30X read depth using the Illumina MiSeq platform (see **Table S1** for details and BioSample accessions).

The same 35 isolates were long-read sequenced in this study. DNA was extracted using the GenFind V3 kit (Beckman Coulter, Indianapolis, United States). Genomic libraries for long-read sequencing were prepared using the Kit12 chemistry and the ligation sequencing kit (SQK-LSK112) in combination with the R10.4 MinION flow cells (n=34), or the Kit9 ligation sequencing kit (SQK-LSK-109) in combination with the R9.4.1 flow cell (n=1) from ONT (Oxford, UK). All libraries were sequenced on the ONT GridION device (GRD-X5G003), aiming for ≥100X read depth. Guppy v6.4.2 was used to basecall and demultiplex the fast5-files with the super accurate basecalling model (see **Table S1** for details).

The 46 isolates used for validation of the ONTmompS *in silico L. pneumophila* SBT approach were ONT sequenced using Kit9/R9.4.1 (n=39) or Kit12/R10.4 (n=7) and basecalled with Guppy v6.4.2 using the super accurate model (see **Table S2** for source details and BioSample accessions).

### 2.3 Assembly and polishing

For the short-reads we used Asmbl v0.2.0 (https://github.com/marithetland/Asmbl) to perform read trimming, *de novo* assembly and quality control: TrimGalore v0.6.7 (https://github.com/FelixKrueger/TrimGalore) was used to remove adapter-contamination and low-quality reads, prior to assembly. We generated two sets of short-read assemblies (for all 35 genomes): The first with Unicycler v0.5.0 (Wick et al., 2017), which uses SPAdes v3.15.5 (Bankevich et al., 2012) for assembly, and the second with Unicycler v0.4.8, which uses SPAdes v3.13.0, includes read error correction and short-read polishes the assembly with Pilon v1.24. In addition, we created assemblies with the following tools and parameters: 1) Unicycler v0.4.8 with SPAdes v3.13.0 without read error correction, 2) without Pilon short-read polishing, and 3) without both, 4) Unicycler v0.5.0 with SPAdes v3.13.0, 5) SPAdes v3.15.5 on its own, 6) SPAdes v3.13.0, and 7) SKESA v2.4.0 (Souvorov et al., 2018).

For the long-reads, Filtlong v0.2.1 (https://github.com/rrwick/Filtlong) was used to discard the worst 5% of read bases and any reads shorter than 1 kbp prior to assembly. We then generated long-read assemblies with three different tools: Unicycler v0.5.0 (which uses a miniasm v0.3-r179 + racon v1.5.0 pipeline), Flye v2.9 (Kolmogorov et al., 2019), and Trycycler v0.5.3 (Wick et al., 2021). Trycycler was run according to the instructions at https://github.com/rrwick/Trycycler/wiki/How-to-run-Trycycler. In short, the reads were subsampled into 12 read-sets and assembled using Flye v2.9 (Kolmogorov et al., 2019), miniasm v0.3-r179 (Li, 2016) + minipolish v0.1.3 (Wick and Holt, 2019) and raven v1.8.1 (Vaser and Šikić, 2021), providing a total of 12 assemblies per isolate, four from each assembly method. The assemblies were then clustered and reconciled. Next, a multiple sequence alignment using MUSCLE v3.8.1551 (Edgar, 2004) was performed, followed by partitioning of the reads. Lastly, a single consensus assembly was generated for each isolate. To correct any small-scale errors, the final assemblies were polished with the long-reads using Medaka v1.7.2 with model r1041_e81_sup_g610 for R10.4 flow cells and model r941_min_sup_g507 for R9.4.1 flow cells, respectively (https://github.com/nanoporetech/medaka).

To assess if ONT-only sequencing with the Kit12/R10.4 chemistry/flow cells was as accurate as the current gold-standard of using ONT data in hybrid with Illumina reads, and thus sufficient for *L. pneumophila* SBT, we also generated long-read first hybrid assemblies. This was done by further polishing the Trycyler+Medaka assemblies with short-reads using Polypolish v0.5.0 and Polca v4.0.5.

### 2.4 Sequence-based typing and identification of *mompS1* and *mompS2*

Sanger sequencing was the method used for SBT at the National Reference Laboratory for *Legionella* until August 2019, when it was replaced by Illumina short-read WGS. All isolates in this study (n=35) were analyzed using Sanger sequencing according to the standardised protocol of the ESCMID Study Group for Legionella Infections (ESGLI) (Gaia et al., 2005; Ratzow et al., 2007) or the ST of the isolate was previously confirmed as part of an External Quality Assessment (EQA) programme.

For the Illumina sequenced isolates, the STs were determined with legsta v0.5.1 (https://github.com/tseemann/legsta) and/or with BLASTn v2.12.0+ (Camacho et al., 2009). Presence of non-identical copies along with the confirmation of *mompS2* were identified by examination of the assembly sequence. For investigations of the *mompS* alleles in STs 154 and 574, we created core genome alignments with RedDog v1b11 (https://github.com/katholt/RedDog) against hybrid assemblies of these STs and viewed the bam files in IGV v2.15.2 (Robinson et al., 2011).

To identify both *mompS1*/*mompS2* and the *L. pneumophila* ST from ONT-only and hybrid assemblies, we have developed ONTmompS v2.0.0 (https://github.com/marithetland/ONTmompS), see section 3.3 for details.

## 3 Results

### 3.1 Challenges with identifying *mompS2* for use in SBT from short-read sequencing data

In our in-house sequence database of *L. pneumophila* genomes (n=233), there were 62 unique STs. Of these, 18 STs (29%) carried non-identical copies of *mompS*. We included a set of 35 isolates in this study; 27 isolates representing each of the 18 STs with non-identical *mompS* copies and 8 isolates (7 STs) with identical copies. Currently, to identify *L. pneumophila* SBT at the National Reference Laboratory for *Legionella*, isolates are subjected to Illumina short-read sequencing, followed by Unicycler assembly (which uses SPAdes), and legsta, which is an *in silico* SBT tool, to determine the ST. We have encountered three main challenges with this method: 1) erroneous calling of the *mompS1* allele instead of *mompS2* by legsta, 2) misassembly of the *mompS* genes when using Unicycler v0.4.8, and 3) failure to resolve the structure of the *mompS*-region with Unicycler v0.4.8 and v0.5.0.

The first challenge occurs when an isolate contains non-identical copies of the *mompS* gene. Upon repeated runs of legsta v0.5.1 using the same input assembly-files, the *mompS2* allele called varied between the two alleles found in *mompS1* and *mompS2*. This was the case for all genomes with non-identical *mompS* copies where both alleles were defined in the database. This issue was solved by examining the assembly graph in Bandage (Wick et al., 2015) and using the *mompS2* flanking sequence in BLASTn searches to identify the correct *mompS2* allele for use in the SBT scheme.

The second challenge is more demanding as it is due to misassembly of the reads in the *mompS* region, i.e. some reads that belonged to *mompS2* assembled into *mompS1* and vice versa. This challenge applies to at least genomes belonging to ST154 and ST574 when using Unicycler v0.4.8 (**Table 1**). Both these STs carry *mompS1* allele 7 and *mompS2* allele 15, differing by 1 nucleotide at position 63, between A and G. For the isolates belonging to these STs (n=8), Sanger sequencing always determined the *mompS2* gene with allele 15, while the short-read assembly often determined the *mompS1* and *mompS2* genes as identical, either with allele 7 or 15. Further, the identified *mompS2* allele sometimes varied between 7 and 15 when the same isolate was re-sequenced. We investigated this with read mapping, which showed that the two nucleotides were mapped to both *mompS1* and *mompS2* at different frequencies. Further, for some of the ST154 and ST574 isolates, the short-read assemblies contained an additional copy of the *mompS* gene, resulting in three identical *mompS* copies per isolate. This misalignment and misassembly of reads occurs because of the repetitiveness of the *mompS*-region and that the Illumina read lengths are shorter (≤300 bp) than the 352 bp part of the *mompS2* gene that is used for allele determination (Gaia et al., 2005).

**Table 1:** Comparison of *L. pneumophila* SBT results for Sanger, Illumina short-read (assemblers with highest and lowest accuracy are shown), ONT-only and hybrid assemblies for genomes in the dataset.

Sanger sequencing or EQA		Illumina WGS (Unicycler v0.4.8, SPAdes v3.13.0)			Illumina WGS (Unicycler v0.5.0, SPAdes v3.15.5)			ONT-only WGS (Trycycler v0.5.3 + Medaka v1.7.2) and hybrid assemblies^a^			Nucleotides difference	Number of isolates
ST	*mompS2*	ST	*mompS1*	*mompS2*	ST	*mompS1*	*mompS2*	ST	*mompS1*	*mompS2*	*mompS 1/2*	
Short-read challenge 2: Misassembly of reads to the *mompS* copies												
154	15	154-1LV/154	7/15	7/15	-	–	–	154	7	15	1	4
574	15	574-1LV/574	7/15	7/15	-	–	–	574	7	15	1	4
1973	15	15	15	15	-	–	–	1973	103	15	3	1
Short-read challenge 3: Untypeability due to too short contigs												
15	26	15	3	26	-	–	–	15	3	26	1	1
20	2	20	93	2	-	–	–	20^b^	93	2	2	1
62	18	62	33	18	-	–	–	62	33	18	1	2
146	2	146	63*	2	-	–	–	146	63*	2	2	1
222	18	222	–	18	-	–	–	222	63	18	1	1
292	19	292	78	19	-	–	–	292	78	19	2	1
354	14	354	14*	14	-	–	–	354	14*	14	1	1
576	19	576	78	19	-	–	–	576	78	19	2	1
659	18	659	63	18	-	–	–	659	63	18	1	1
864	55	864	55*	55	-	–	–	864	55*	55	8	1
1328	46	1328	46*	46	-	–	–	1328	46*	46	1	1
2118	1	2118	10*	1	2118	–	1	2118	10*	1	9	1
2923	21	2923	9*	21	-	–	–	2923	9*	21	3	2
3138	2	3138	63	2	-	–	–	3138	63	2	1	1
3140	41	3140	41*	41	-	–	–	3140	41*	41	3	2
Controls: STs that were typeable with short-reads												
1	1	1	1	1	-	–	–	1	1	1	0	1
68	14	68	14	14	68	14	14	68	14	14	0	2
2110	13	2110	13	13	2110	13	13	2110	13	13	0	1
2630	12	2630	12	12	2630	12	12	2630	12	12	0	1
2454	9	2454	9	9	2454	9	9	2454	9	9	0	1
1324	6	1324	6	6	1324	6	6	1324	6	6	0	1
3142	13	3142	13	13	3142	13	13	3142	13	13	0	1

a The results from ONT-only (Trycycler+Medaka) and hybrid assembly (Trycycler+Medaka+Polypolish+Polca) were identical.

b Sequenced using Kit9 chemistry and R9.4.1 flow cell.

SBT, sequence-based typing; ONT, Oxford Nanopore Technologies; EQA, external quality assessment; WGS, whole-genome sequencing; ST, sequence type.

Inability to determine a mompS allele and thus the ST is marked by “–”; untypeability of the ST is designated with the closest matching ST as “ST-nLV”; “*” marks the closest matching allele if <100% and ≥ 90% identity; “?” marks the closest matching allele if <100% and ≥ 80% coverage.

The third challenge; failure to resolve the structure of the *mompS*-region, which resulted in untypeability of isolates, was seen for 27 isolates when using Unicycler v0.5.0, including the eight that were misassembled with Unicycler v0.4.8 (see **Table 1**). Of these, 26 genomes carried non-identical *mompS* copies and one genome (ST1) had identical *mompS* copies. Investigations of the assembly graphs showed that the *mompS*-region of these isolates consisted of several short contigs that did not span the *mompS* genes and the flanking regions, which are used to distinguish *mompS2* from *mompS1.*

We investigated whether the read length or read depth affected the results we observed with the short-read assemblers. The isolates were sequenced with different Illumina kits leading to paired-end read lengths of 2x300 bp, 2x250 or 2x150 bp (see **Table S1**); we observed no pattern indicating that either kit was linked to typeability. Similarly, we saw no link between the read depth and typeability (the read depths ranged 32-212X). As there were differences in the short-read assemblies with the two Unicycler versions, we tested several methods of assembly with/without polishing and read error correction to see if any would consistently type the ST of all the genomes (see section 2.3). Unfortunately, none of the methods consistently typed all the isolates, however those that included short-read polishing did produce better results (for details see **Table S3**).

In sum, the observed misassembly or untypeability of *mompS2*, which varied with different assemblers or versions of the same tools, indicates major challenges with using short-read sequencing to identify the *mompS2* allele, and thus the ST, for several *L. pneumophila* isolates.

### 3.2 Long-read sequencing resolves the *mompS*-region and provides the necessary accuracy for *L. pneumophila* SBT

**Figure 2:**
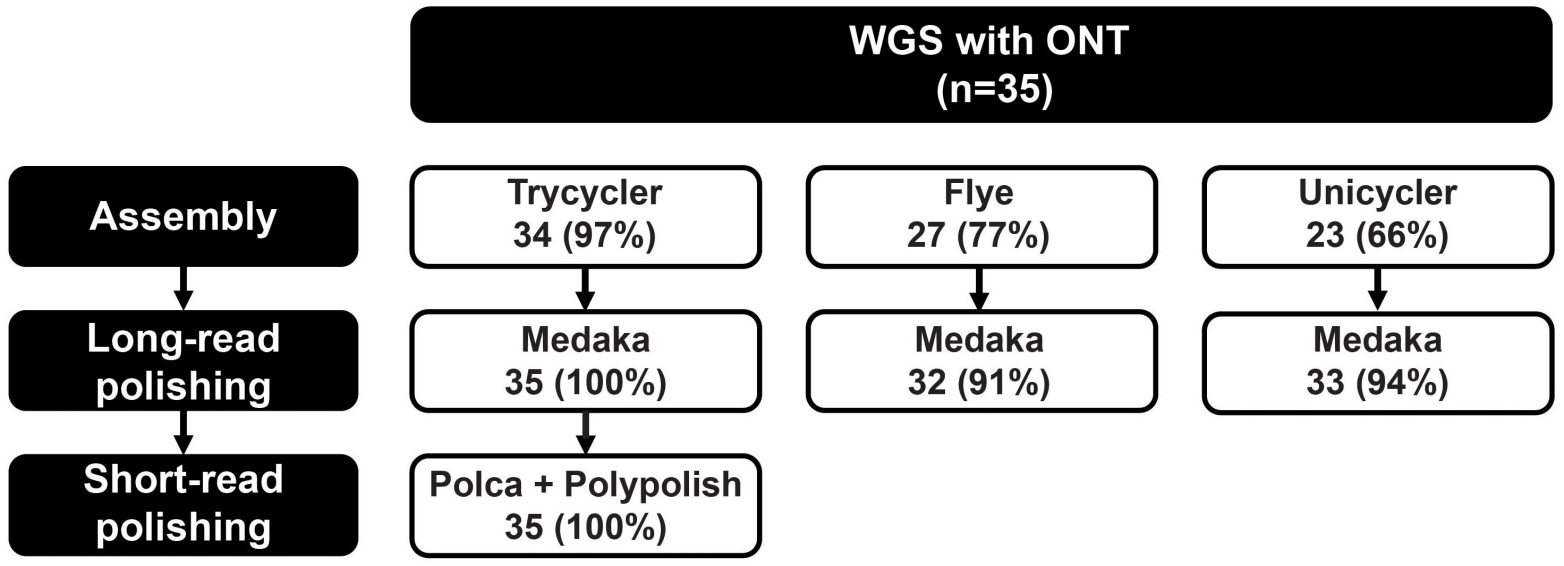
Accuracy of *Legionella pneumophila* ST calls using ONT-only or hybrid assemblies. Three assemblers (Trycycler v0.5.3, Flye v2.9, Unicycler v0.5.0) were used for long-read only assembly, followed by long-read polishing with Medaka v1.7.2. The Trycycler+Medaka assemblies were further short-read polished with Polca v4.0.5 and Polypolish v0.5.0 to create hybrid assemblies. Genomes were sequenced with Kit12/R10.4 (n=34) and Kit9/R9.4 (n=1). The number (and percentage) in each box indicates the amount of isolates whose ST was accurately called when using these tools. See also **Table S1** for more details.

The combination of long-reads spanning the entire *mompS*-region (**Figure 1**) with the recent improvements in accuracy to the ONT sequencing technology, led us to the hypothesis that ONT reads on their own would be sufficient for calling *mompS* and subsequently *L. pneumophila* SBT. We assessed three common methods of long-read assembly (Trycycler, Flye and Unicycler) to see if *mompS2* could be reliably identified using ONT-only reads. The 35 isolates were ONT sequenced, assembled and polished to create seven sets of assemblies for each isolate (**Figure 2**).

The *mompS*-region of all 35 isolates was resolved using either of the methods, but only Trycycler+Medaka and hybrid assemblies with long and short-read polishing assigned the correct ST in accordance with Sanger/EQA results in all isolates (**Figure 2**). For the Trycycler-only assemblies, one genome had an incorrect *proA* allele, which was corrected with Medaka long-read polishing. For the Flye+Medaka and Unicycler+Medaka assemblies, three and two genomes had one incorrect allele call, respectively.

To perform *in silico* SBT from long-read assemblies we first ran legsta, but this tool gave inconsistent results similar to what we observed with short-reads: some alleles were not called, even though we could identify them with BLASTn searches (most commonly *flaA* allele 11 and *neuA* allele 11), and the *mompS* allele was sometimes incorrectly called in genomes with non-identical copies.

### 3.3 ONTmompS for *in silico* SBT of *L. pneumophila* from long-reads

To identify the *mompS1* and *mompS2* alleles and the *L. pneumophila* ST from ONT-only and hybrid assemblies, we have developed the open source tool ONTmompS v2.0.0 (https://github.com/marithetland/ONTmompS). For each input assembly, the tool first identifies the *mompS2* allele by distinguishing it from *mompS1*: BLASTn v2.12.0+ is used to query the assembly against a 987 bp reference sequence (see **Figure 1**). This reference was originally defined for use in the *mompS* tool (https://github.com/bioinfo-core-BGU/mompS) developed by Gordon et al. (2017), and is a conserved sequence that covers the 352 nucleotides of the *mompS2* gene that are used for allele definition, and flanking regions, including the 430F upstream primer and the downstream primer 1116R. Next a pairwise Smith-Waterman local sequence alignment (EMBOSS v6.6.0.0) (Smith and Waterman, 1981) is performed on each *mompS* copy against the 1116R primer sequence, which is the downstream primer traditionally used in Sanger sequencing and which can be used to distinguish *mompS2* from *mompS1* (Gaia et al., 2005; Gordon et al., 2017). The copy that aligns to the primer is assigned as *mompS2* and the other copy as *mompS1*. Once the *mompS2* copy has been identified, the alleles of all seven genes in the SBT scheme and the resulting ST are determined using the same logic as that developed for *in silico* multi-locus sequence typing (MLST) of *Klebsiella pneumoniae* in Kleborate v2.3.1 (https://github.com/katholt/Kleborate) (Lam et al., 2021). ONTmompS reports two main results: 1) the allele numbers of the *mompS1* and *mompS2* copies and 2) the ST together with the alleles of the seven genes in the scheme.

Using ONTmompS v2.0.0, we were able to identify *mompS2* (and thus ST) for all Trycycler+Medaka and hybrid assemblies, in accordance with Sanger sequencing or EQA results for all 35 isolates.

This analysis showed that all the isolates that were misassembled or untypeable when using short-read assembly methods were typeable with ONT-only assemblies when using Trycycler for assembly together with Medaka long-read polishing. The controls, i.e. the isolates that had identical *mompS* copies and were typeable with short-read sequencing, were also typeable with this ONT-only workflow.

To further test and confirm that ONT-only reads are sufficient for *L. pneumophila* SBT, and potentially also from the Kit9/R9.4.1 chemistry/flow cells, we tested our workflow on an additional 46 ONT-sequenced isolates (**Table S2**). Of these isolates, 39 were sequenced using Kit9 chemistry/R9.4.1 flow cells, the remaining seven with Kit12/R10.4. All isolates were basecalled with the super accurate model and assembled with Trycycler+Medaka followed by analyses using ONTmompS. The *mompS*-region was resolved for all the isolates and STs were typeable and in accordance with Illumina and Sanger sequencing where that was available. This indicates that ONT-only reads (from at least Kit9/R9.4.1 and Kit12/R.10.4) are a reliable and effective solution for *L. pneumophila* SBT.

## 4 Discussion and conclusion

In this study, we encountered several challenges when calling *mompS2* from Illumina short-read sequences and have shown that ONT long-read sequences are a reliable alternative for determining *mompS2* and the ST of *L. pneumophila*. Today, epidemiological investigations of *L. pneumophila* and associated disease or outbreaks are mainly done by Sanger SBT and/or WGS analyses. The challenges with identifying *mompS2* due to non-identical copies of *mompS* and the short length of Illumina reads is a hindrance for routine use of short-reads in typing and surveillance. In 2015, a WGS-based cgMLST typing scheme for *L. pneumophila* was proposed, which showed high resolution of strains within the same ST/clonal complex from short-reads, but the challenge with typing due to non-identical *mompS* copies remained unsolved (Moran-Gilad et al., 2015). In 2017, the mompS tool (https://github.com/bioinfo-core-BGU/mompS) was developed to solve the challenge by accepting only reads that also flanked the *mompS2* gene and thus ensuring correct allele calls, and to ensure backwards compatibility with the Sanger SBT scheme (Gordon et al., 2017). However, in our experience the tool often failed, due to too low read coverage of the 352 bp *mompS2* sequence once the reads that do not overlap the gene and a flanking region have been filtered out. It is therefore not included as part of our standard bioinformatic analyses for *L. pneumophila* typing. In 2018, the first release of legsta was published (https://github.com/tseemann/legsta). This *in silico* approach for *L. pneumophila* SBT takes assemblies as input and uses the *L. pneumophila* SBT database together with primer sequences to identify the correct alleles for the SBT scheme. For assemblies from short-reads, long-reads, and hybrid assemblies, we unfortunately experienced that this tool was sometimes unable to call alleles or called the incorrect *mompS* allele. Neither the mompS tool nor legsta have been maintained or updated for several years.

Our analyses of short-read *L. pneumophila* genomes also showed that different assemblers or even different versions of the same assembler affected the typeability of the *mompS2* allele, adding yet another layer of complexity to the challenge of identifying STs from short-reads. When taking together the challenges of resolving the *mompS*-region from short-reads and the issues with identifying the *mompS2* allele with existing tools, it was clear that an alternative method was needed, which is why we explored using ONT long-reads and developed ONTmompS to perform *in silico L. pneumophila* SBT on ONT-only or hybrid assembled genomes.

With ONT-only reads and the ONTmompS tool, we were able to resolve the *mompS-*region and to perform *in silico L. pneumophila* SBT on all 81 genomes that were tested (the initial 35 genomes + the 46 that were used for validation). Both the Kit9/R9.4.1 and Kit12/R10.4 chemistry/flow cells with Guppy’s super accurate basecalling model were used. The assemblies that were created with Trycycler followed by long-read polishing with Medaka (Trycycler+Medaka) were assigned the same ST as with Sanger SBT, EQA or hybrid assemblies for all isolates where these results were available, and an ST was assigned when using ONTmompS in all of the tested genomes. With Trycycler-only, Flye+Medaka and Unicycler+Medaka, the STs of a few of the 35 genomes did not match with the Sanger/EQA or hybrid assemblies. There were no apparent quality issues with these genomes. We therefore recommend using Trycycler with Medaka long-read polishing for ONT-only assemblies for *in silico* SBT of *L. pneumophila*.

There was no difference in the typeability of the genomes that were ONT sequenced with Kit9/R9.4.1 and Kit12/R10.4, indicating that both chemistries/kits are appropriate for determining STs of *L. pneumophila*. This is consistent with two recent studies of ONT sequencing: Wagner et al. (2023) showed that genomic analyses of *Bordetella pertussis* from ONT-only assemblies from Kit12/R10.4 yielded results with comparable accuracy as from short-reads. Similarly, Foster-Nyarko et al. (2023) found that ONT-only reads from Kit10/R9.4, basecalled with the super accuracy model, with or without Medaka long-read polishing, were sufficient for calling ST, capsule type and AMR determinants for *Klebsiella pneumoniae*, but not sufficient for defining outbreak clusters. It was outside the scope of our study to investigate if the R10.4 ONT-only reads could be used for defining outbreak clusters of *L. pneumophila*. However, Sanderson et al. (2023) recently compared the raw read accuracy and assembly accuracy of ONT Kit10/R9.4, Kit12/R10.3, Kit12/R10.4, Kit12/R10.4 with duplex reads, and Illumina sequencing of four bacterial pathogens (not *L. pneumophila*). Sereika et al. (2022) did a similar comparison. Both studies found that ONT Kit12/R10.4 duplex reads that were basecalled with the super accuracy model could be used for complete reconstruction of bacterial genomes without the use of Illumina reads. However, Sanderson et al. (2023) noted that recovery of small plasmids was inconsistent and that hybrid assemblies still remain the most cost-effective and robust approach for bacterial whole-genome reconstruction.

By combining real-time basecalling and the ONT Kit12/R10.4, Wagner et al. (2023) recently showed that it is possible to perform highly accurate and fast high-resolution typing of bacterial pathogens while sequencing is ongoing, highlighting the time-saving potential of the ONT-technology in outbreak situations. Furthermore, the flexibility in the number of samples and/or flow cells that can be run on for instance ONT’s GridIon device, makes ONT well-suited and efficient for analyzing both a single isolate and larger collections, e.g. in suspected outbreaks. In our laboratory, the cost of generating ONT-only assemblies is overall lower than for Illumina-only assemblies, with the difference being more profound for lower sample numbers. However, the cost per isolate for both technologies are dependent on the kit used, number of samples analyzed and how well the capacity of the sequencing kit/flow cell is utilized. ONT has recently launched Kit14/R10.4.1 flow cell promising an even more improved accuracy. Given our results with Kit9 and Kit12 we expect to obtain similar results with Trycycler+Medaka and hopefully even better results with the other assembly methods with the improved ONT technology.

*L. pneumophila* SBT is important for the characterization of *L. pneumophila* isolates and for standardized comparison of results over time and between different laboratories (Raphael et al., 2016). In source investigations, the SBT serves well to identify potential sources in the initial phase of the investigation. However, due to the lower discriminatory power of this method compared to whole genome genetic relatedness analyses, further analyses are usually needed to confirm a potential source (Raphael et al., 2016; Ricci et al., 2022). By utilizing long-read WGS, SBT can easily be assessed in an initial analysis, and in-depth whole-genome analyses may also be performed, either together with complementary short-reads to correct ONT read errors, or ONT reads on their own if and when these have been proven sufficient to use in whole genome analyses, for which evidence is starting to emerge (Sereika et al., 2022; Sanderson et al., 2023; Wagner et al., 2023).

To conclude, ONT-only sequencing is sufficient for identifying *mompS2* and *L. pneumophila* ST. Our analyses show that ONT-only assemblies provide a cost- and time-efficient solution for determining *L. pneumophila* ST from WGS, where Illumina short-reads often fail to identify *mompS2*. For the best results, we recommend basecalling with Guppy using the super accurate basecalling model, assembly with Trycycler and polishing with Medaka, before identifying the ST with ONTmompS.

## Data availability statement

The datasets presented in this study are available online in the European Nucleotide Archive under accession numbers PRJEB58776 and PRJEB50383. Please see **Tables S1** and **S2** for individual accession numbers.

## Author contributions

AK, MH, EB, and IL conceptualized the study. AK and EB performed genome sequencing. MH and MS developed ONTmompS. AK and AB analyzed Sanger and WGS results. IL provided resources and supervision. AK, MH and EB wrote the first draft of the manuscript. All authors contributed to data interpretation, reviewed and edited the manuscript, and have read and agreed to the published version of the manuscript.
